# 3D structure reconstruction of nanoengineered polymeric capsules using Coherent X-Ray diffraction imaging

**DOI:** 10.1016/j.mex.2021.101230

**Published:** 2021-01-13

**Authors:** S. Erokhina, L. Pastorino, D. Di Lisa, A.G. Kiiamov, D.A. Tayurskii, S. Iannotta, V. Erokhin, A.R. Faizullina

**Affiliations:** a Institute of Fundamental Medicine and Biology, Kazan Federal University 420012 Kazan, Russia; b University di Genoa 16126 Genoa, Italy; c Institute of Physics, Kazan Federal University 420008 Kazan, Russia; d IMEM-CNR Istituto dei Materiali per l'Elettronica ed il Magnetismo – Consiglio Nazionale delle Ricerche 43123 Parma, Italy

**Keywords:** Coherent X-ray diffraction imaging, Nanoengineered polymeric capsules, Gold nanoparticles

## Abstract

Nanoengineered polymeric capsules (NPCs) are smart objects that can be filled in with some desired chemical substance. They are considered among the most versatile tools in biology, pharmacy, medicine etc. Most often they have been used as containers for drug delivery. Main tools for studying their structure are electron (SEM, TEM) and fluorescence microscopies. In the case of electron microscopies, the main peculiarity was connected to the necessity of dried samples usage. In the case of fluorescence microscopy, the possible resolution is restricted by diffraction limits. The natural environment of the NPCs is liquid medium. In this paper we have developed a method of NPCs’ structure investigation in liquid medium using coherent X-ray diffraction imaging (CXDI). The main points of this article are summarized as:

• The procedure of NPCs’ synthesis using layer-by-layer technique including gold nanoparticles;
• Coherent X-ray diffraction imaging of the samples in liquid medium;
• Imaging of objects without freezing of the sample.

## Graphic abstract

**Figure:**
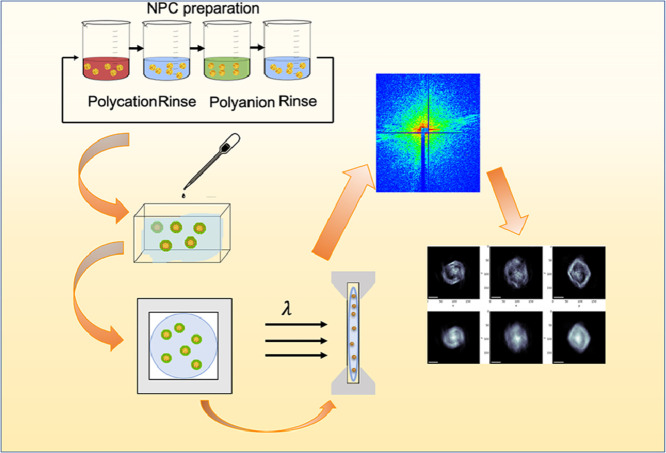

**Specifications Table**

**Table:** 

**Subject Area**	Pharmacology, Toxicology and Pharmaceutical Science
**More specific subject area**	Nanoengineered polymeric capsules (NPCs); layer-by-layer technique; coherent X-ray diffraction imaging (CXDI).
**Method name**	3D reconstruction of NPCs using Coherent X-Ray diffraction imaging
**Name and reference of original method**	1. Marchesini, S., et al. "Coherent X-ray diffractive imaging: applications and limitations." Optics Express 11.19 (2003): 2344-2353. 2. Shapiro, David, et al. "Biological imaging by soft x-ray diffraction microscopy." Proceedings of the National Academy of Sciences 102.43 (2005): 15343-15346.
**Resource availability**	Report of the experiment SC-4544 on ESRF website http://ftp.esrf.fr/pub/UserReports/83683_B.pdf

## *Method details

 

## Introduction

Nanoengineered polymeric capsules (NPCs) are smart micro- and nano containers which are widely used as a container in the field of drug delivery and targeted release of pharmaceutical substances[1]. One of the main techniques for their synthesis is layer-by-layer technique. For this reason, different polyelectrolytes are assembled as a shell on a template with the successive dissolving of it [2,3]. The diameter of a capsule is from 50 nm till several microns. These hollow containers can be filled in with a various chemical substance, for example with a drug. Also, one of the specificities of such containers is that the shell can be functionalized with an application of different polymers, enzymes, nanoparticles, inorganic substances etc. Significant characteristic of NPCs is the ability to vary the permeability of a shell by pH, temperature, magnetic field, UV, light, etc., connected to the opening or closing of pores in the capsule's shells [4,5].

The easiest and most studied method to vary the capsules shell permeability is connected to the pH variation of the solution, containing capsules. The pores of the capsules are closed when pH of the solution is more than 8 and opened at pH less than 4.5. When pH of the solution is neutral, both opened and closed capsules can coexist in the solution.

Up to now the structure of capsules was studied mostly for dried samples, using electron and scanning probe microscopies (SEM, TEM, AFM [6]). Fluorescence microscopy was used for investigation of capsules structure in liquid medium. However, it requires the use of fluorescence probes, that can disturb the original structure of the objects. Moreover, the resolution of the method is restricted by the diffraction limits. To sum up, all these methods were not able to provide a detailed structure of the NPCs in their natural liquid environment.

On the other hand, there is a powerful tool, coherent X-ray diffraction imaging (CXDI), allowing the reconstruction of 3D structure of samples with 10 nm resolution, including the distribution of the matter not only at the surface, but also in the core of objects. Of course, the sample itself must have sufficient contrast of electron densities between its different components [7], [8], [9]. The illumination of the sample with X-ray beam results in the scattering pattern, which is acquired by the detector for the structure reconstruction.

This kind of imaging can be done only if two important requirements are satisfied: coherent X-ray beam must be used and its intensity must be rather high. The first requirement is necessary for obtaining diffraction patterns, while the second one allows to work with shorter time, necessary for the diffraction pattern acquisition at each inclination angle with respect to the incident beam, what results in rational duration of the experiment. Both requirements can be satisfied only by using synchrotron radiation. ID 10 station of ESRF is one of the most appropriated places, where these experiments can be done.

CXDI had been used to analyze different types of samples. Bioorganic samples were previously studied in frozen state [10].

CXDI is a method which is based on diffraction. Image reconstruction is performed by the inverse Fourier transformation in the case when the diffracted wave field of the sample is known at the far field. However, during the experiment, only the amplitude of the wave field is available, the phase is not measured. According to Sayre, selection of the diffracted amplitude at the Nyquist frequency of the intensity pattern can recode phase information into data [11]. Using this data, phase search algorithms set a priori known information in the form of issues iteratively to seek a solution [10,12,13].

## Method

**Fig. 1:**
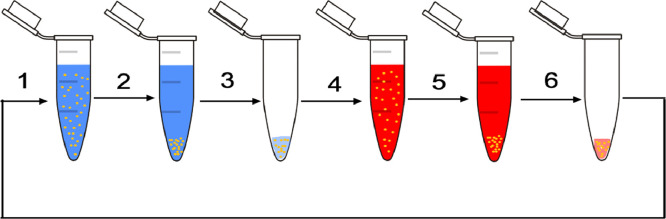
Scheme of shell formation. 1) PSS was added to CaCO_3_ microparticles. 2) Centrifugation. 3) Supernatant is removed. 4) Adding of PAH. 5) Centrifugation. 6) Supernatant is removed. These steps are repeated till the required number of layers are received.

Nanoengineered polymeric capsules were prepared according to [14,15] with minor alterations. Calcium carbonate CaCO_3_ microparticles were prepared by adding of 1 ml 0.33 M sodium carbonate (Na_2_CO_3_) to 1 ml 0.33M calcium chloride (CaCl_2_). The microparticles were centrifuged and washed three times in ultrapure water. There were prepared two types of capsules. In the first case, the shell contained 3 bilayers of poly(sodium 4-styrenesulfonate) (PSS) (2 mg/ml) and poly(allylamine hydrochloride) (PAH) (2 mg/ml), in the second case the – 6 bilayers. The scheme of layer-by-layer technique for the capsule formation is represented on Fig. 1.

Hollow polyelectrolyte capsules were fabricated in 0.15M NaCl solution by alternating adsorption of PSS/PAH layers onto CaCO_3_ microparticles. After the shell formation, templates were dissolved at pH 6 in ethylenediaminetetraacetic acid (EDTA) solution. Microcapsules were suspended in 1 ml of EDTA (0.2 M) for 5 min, centrifuged and washed 3 times with ultrapure water. 1 ml of CdCl_2_ (0.125M) was added to each type sample and stored overnight at 4 °C. This was done in order to increase the contrast of the objects. Then, microcapsules were centrifuged, washed (three times) and stored at 4 °C. pH values of 4.0 and 9.0 were used for opening pores, loading of gold nanoparticles and closing pores in microcapsules, respectively. The loading was performed by exposing the hollow microcapsules to a dispersion of gold nanoparticles (1 mg/ml), for 24 h with continuous shaking.

**Fig. 2:**
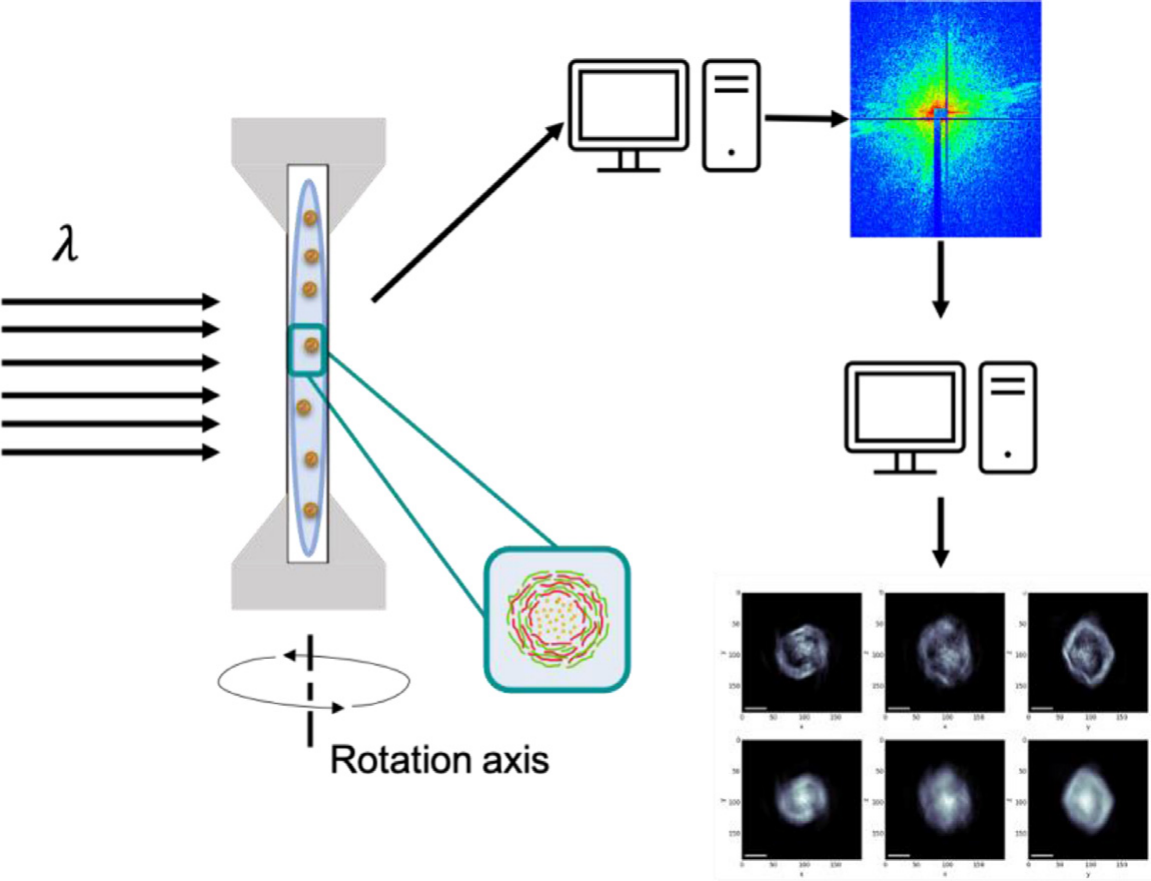
Scheme of the experiment: NPC solution is placed between 100-nm-thick silicon nitrate membranes, during the acquisition it is rotated around the axis and shifted along the height for the position adjustment. Diffraction pattern were acquired at each rotation angle. Reconstruction of the capsules structure was done according the protocol on ID-10 beamline (ESRF).

Liquid solution of capsules was placed between two silicon nitrate windows. The sizes of the silicon nitrate windows (Silson, UK) were: frame: 10 mm x 10 mm; membrane: 3 mm x 2 mm; thickness – 100 nm. These windows were assembled in a special frame, fabricated using 3D printer. 10 µl drop of diluted (1:10) solution of capsules, provided correct dispersion of objects, was placed inside the chamber for the acquisition. In order to prevent the evaporation of the liquid medium, the whole perimeter of the double membrane system was covered by the “vacuum grease”. Schematically it is shown in Fig. 2.

Coherent X-ray diffraction imaging experiments were done at ID10 station of European Synchrotron Radiation Facilities (ESRF, Grenoble) [16,17], all capsules shells were decorated with cadmium (II) chloride for better contrast of the samples [18]. 8.1keV radiation was used for the imaging. The sample–detector distance was 5280 mm. The scheme of the experiment is presented on the Fig. 2.

Samples were rotated relatively to the incident X-ray beam in the range of –70° to 70°.Scattering patterns were acquired after each 0.05°. Data acquisition at each point was 1 s. During the acquisition, the position of the frame was shifted vertically relative to the beam position. This was done in order to acquire scattering patterns exactly in the position where the investigated capsule is in a certain moment. Since the sample is in the liquid phase, the capsules descend in time from the upper point of the membrane to its bottom due to the gravity. Therefore, the vertical position of the frame with respect to the incident beam was adjusted after the acquisition of every 50 scattering curves. The range of the frame rotation around the axis was selected for each sample.

**Fig. 3:**
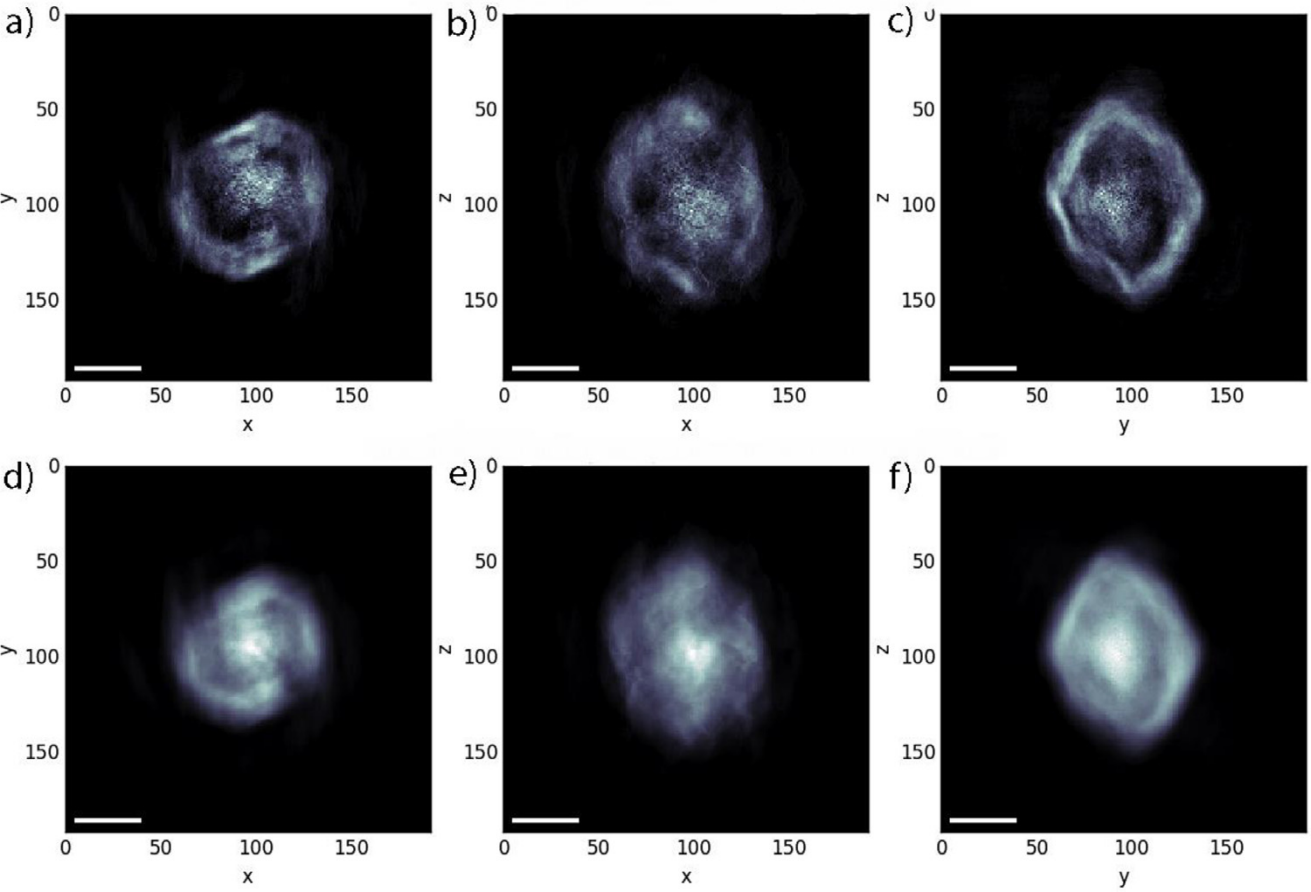
Reconstructed images of 12 PSS/PAH layers NPC at pH 6.5 in liquid medium: a), d) top views; b), e) front views; c), f) side views for the cross section (a, b, c) and the plane projection (d, e, f).

As a result, we have received diffraction images which were processed by the specific protocol, established on the ID10 station [19]. The reconstructed images of the diffraction patterns are presented on Fig. 3. Images a, b, c represent cross section, while images d, e, f represent plane projection for top, front, and side view.

## Method validation

For the first time the images of NPCs in liquid medium studied by CXDI were received. Their reconstruction has demonstrated the explicit difference of the structure depending on whether capsules were under dry conditions or in a liquid medium [20]. In addition, this reconstruction has proved the shape of capsules which was suspected as a spherical one. Moreover, it is possible to investigate not only the external part of capsules but their inner part. The addition of gold nanoparticles enhances the contrast.

The reconstruction has proved the spherical shape of capsules and has shown their structure in three projections: front, top and cross-section. 3D movie of the reconstructed capsule structure is presented in supplementary materials.

Withal, the permeability of the shell can be studied by CXDI. As a sample we have used a solution of capsules under different pH. In this case it was possible to monitor changes in the permeability of the shell by altering the medium of the capsules’ solution. In this work we have studied objects under pH of 4.0, 6.5 and 8.5. These values of pH are related to the opened capsules, both possible states and closed capsules respectively.

When pH was 4.0, the gold nanoparticles were in the entire volume of the capsule, we conclude that the pores of NPCs are opened, and the nanoparticles can move in the total volume. At pH 6.5 some nanoparticles were in the shell, some in the center of the capsules, so we can suggest that the pores are closing, but anyway some of them are still open. At pH 8.5 all gold nanoparticles are concentrated in the center of the capsule. That can indicate that the pores are closed.

Accordingly, analyzing this dependence, changes in the permeability of the shell can be explained by a sharp transition regarding the shell properties as a function of pH.

Supplementary material *and/or* Additional information:

## Declaration of Competing Interest

The authors declare that they have no known competing financial interests or personal relationships that could have appeared to influence the work reported in this paper.

## Appendix Supplementary materials

Image, video 1
